# Subtyping Options for *Microsporum canis* Using Microsatellites and MLST: A Case Study from Southern Italy

**DOI:** 10.3390/pathogens11010004

**Published:** 2021-12-22

**Authors:** Chioma Inyang Aneke, Adéla Čmoková, Vít Hubka, Wafa Rhimi, Domenico Otranto, Claudia Cafarchia

**Affiliations:** 1 Dipartimento di Medicina Veterinaria, Università degli Studi Aldo Moro, 70010 Bari, Italy; chioma.aneke@unn.edu.ng (C.I.A.); domenico.otranto@uniba.it (D.O.); 2 Department of Veterinary Pathology and Microbiology, University of Nigeria, Nsukka 410001, Nigeria; 3 Department of Botany, Faculty of Science, Charles University, 12801 Prague, Czech Republic; adela.cmokova@natur.cuni.cz; 4 Laboratory of Fungal Genetics and Metabolism, Institute of Microbiology of the Czech Academy of Sciences, 14220 Prague, Czech Republic; 5 Faculty of Veterinary Sciences, Bu-Ali Sina University, Hamedan 6517658978, Iran; wafa.rhimi@uniba.it

**Keywords:** microsatellite typing, multilocus sequence typing, population structure, genetic diversity, zoonotic infections, zoophilic dermatophytes

## Abstract

*Microsporum canis* is considered one of the most common zoophilic dermatophyte species causing infections in animals and humans worldwide. However, molecular epidemiological studies on this dermatophyte are still rare. In this study, we aimed to analyse the population structure and relationships between *M. canis* strains (*n* = 66) collected in southern Italy and those isolated from symptomatic and asymptomatic animals (cats, dogs and rabbits) and humans. For subtyping purposes, using multilocus sequence typing (MLST) and multilocus microsatellite typing (MLMT), we first used a limited set of strains to screen for variability. No intraspecies variability was detected in six out of the eight reference genes tested and only the ITS and IGS regions showed two and three sequence genotypes, respectively, resulting in five MLST genotypes. All of eight genes were, however, useful for discrimination among *M. canis*, *M. audouinii* and *M. ferrugineum*. In total, eighteen microsatellite genotypes (A–R) were recognized using MLMT based on six loci, allowing a subdivision of strains into two clusters based on the Bayesian iterative algorithm. Six MLMT genotypes were from multiple host species, while 12 genotypes were found only in one host. There were no statistically significant differences between clusters in terms of host spectrum and the presence or absence of lesions. Our results confirmed that the MLST approach is not useful for detailed subtyping and examining the population structure of *M. canis*, while microsatellite analysis is a powerful tool for conducting surveillance studies and gaining insight into the epidemiology of infections due to this pathogen.

## 1. Introduction

*Microsporum canis* is considered one of the most common zoophilic dermatophytes causing infections in animals and humans worldwide [1,2]. The main natural habitat of this species is primarily the furred skin of cats, followed by dogs and horses, where it frequently resides without causing symptoms [3,4]. In Italy, *M. canis* is the dermatophyte that is most frequently isolated (over 80%) from dogs and cats and is a common cause of tinea capitis and tinea corporis in humans, who might acquire those infections after contact with infected animals [3,5]. The identification of the source of infection is an important step to prevent the spread of *M. canis*. An important method of evaluating the source of infection is using sensitive molecular markers that can differ among strains [6]. Typization may also be useful to track recurrence or reinfection after treatment and analyse connections between genotype/lineage and virulence or drug resistance [7]. However, epidemiological studies of *M. canis* infections that include the subtyping of strains remain rare. This is mainly due to a lack of polymorphic molecular markers [3,8] and a predominantly clonal spread and thus low intraspecies variability of this pathogen [9]. Restriction fragment length polymorphisms (RFLP) of mitochondrial DNA genes, random amplification of polymorphic DNA (RAPD) and multilocus sequence typing (MLST) of the internal transcribed spacer (ITS) and nontranscribed spacer (NTS) regions of ribosomal RNA genes (rDNA) have been employed for *M. canis* typing, usually resulting in insufficient differentiation among strains with different geographical provenance or host origin [3,10]. In addition, many of these techniques are obsolete and their utility is frequently constrained by their poor reproducibility [7,8].

Multilocus microsatellite typing (MLMT) is currently one of the most efficient typing tools available for dermatophytes because it is reproducible, easy to perform and suitable for large-scale epidemiological studies due to its advantages in terms of speed and cost. Microsatellites (short tandem repeats of two to six nucleotides) are known to be highly polymorphic and have been widely used for genotyping and studying the population structure of dermatophytes [6,11,12] and other pathogenic fungi (e.g., *Aspergillus* or *Candida*) [13,14]. However, studies on the genotyping and population structure of *M. canis* remain rare or were performed by using a low number of microsatellite markers or strains [3,4].

Although various techniques have been employed for typing *M. canis*, their discriminatory power is usually low, and each has been limited to a few studies. The aims of this study were to evaluate the possibilities of the genotypic characterization of *M*. *canis* strains isolated in southern Italy from different hosts by using (i) an MLST approach involving a total of eight phylogenetic markers commonly used in fungal taxonomy or population genetic studies and (ii) an MLMT approach with both novel microsatellite markers and those previously employed [3,4].

## 2. Results

### 2.1. Multilocus Sequence Typing (MLST)

A total of six target genes (*tubb*, *RPB2*, *tef1-α*, *CaM*, *act*, *gapdh*, *mcm7*) out of the eight showed no intraspecies variability in the test set of eight strains from different hosts/localities. Only ITS and IGS rDNA showed variability between the tested isolates and were successfully amplified in 62 strains. All eight loci were useful for differentiation among *M. canis*, *M. audouinii* and *M. ferrugineum* and the accession numbers for the unique sequences are listed in Table S1.

In total, the MLST approach with two loci identified five combined ITS-IGS genotypes among the 62 strains (Table 1).

The ITS region showed two MLST genotypes differing from each other by a single substitution in the 5.8 S region; genotype ITS-G1 (GenBank accession: LR989561) was identical to the *M. canis* ex-type strain CBS 496.86 (MH861991) and was present in 59 out of the 62 strains. The genotype ITS-G2 (GenBank accession: LR989562) was found in only 3 strains. Three MLST genotypes were found in the IGS region. The most common genotype, IGS-G1 (GenBank accession: LR989270), was detected in 49 strains; IGS-G2 (GenBank accession: LR989271), with a single substitution compared to IGS-G1 (position 837 in the alignment), was present in 11 strains; and IGS-G3 (GenBank accession: LR989272), with a single substitution compared to IGS-G1 (position 838), was present in two strains.

**Figure 1:**
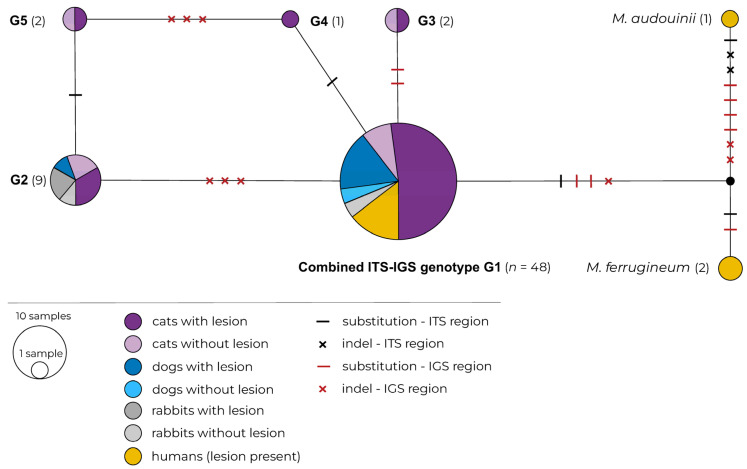
Haplotype network of *Microsporum canis* based on combined sequence data from ITS and IGS regions. Haplotypes are indicated by circles and their sizes correspond to the number of strains sharing this haplotype. Dashes on the connecting lines indicate substitutions and crosses indels; hosts and presence/absence of lesion are indicated by various colours. Strains of *M. ferrugineum* (CBS 497.48, SK 1775/19) and *M. audouinii* (CBS 404.61) were used as outgroups.

The haplotype network of the combined ITS and IGS data with information on the host and the presence/absence of skin lesions is shown in Figure 1.

Strains with the MLST genotype G1 were found in different hosts with and without lesions, while MLST genotypes G3, G4 and G5 were found only in cats. The MLST genotype G2 was found in cats, dogs and rabbits with and without skin lesions. All human isolates were included among strains with MLST genotype G1.

### 2.2. Multilocus Microsatellite Typing (MLMT)

Primer pairs for a total of 13 loci were newly designed and tested together with an additional eight markers from Pasquetti et al. [4]. Invariable loci, loci with interrupted repeats, or loci containing two or more repeat motifs within the fragments (verified by DNA sequencing) were excluded. This led to a final number of six markers with an even distribution in the genome and different lengths (for the purpose of multiplexing). These markers were successfully analysed in 65 *M. canis* strains from southern Italy.

Six markers exhibited polymorphic profiles, with GT_17_C, AG_12_, GT_14_ and CAT_8_ having two alleles each and TC_10_ and GT_17_B having four alleles each. In total, this MLMT scheme resulted in 18 multilocus genotypes (A–R) (Table 1) and the corresponding Simpson’s diversity index was 0.84.

Genotype C was shared by the highest number of strains (*n* = 23), followed by genotypes B (*n* = 8), I (*n* = 8), D (*n* = 7), G (*n* = 3), K, O and P (*n* = 2); the remaining genotypes were found in only one strain each (Table 1). Six genotypes were found in multiple host species, whereas 12 genotypes were found in only one host. In particular, genotypes C and D were isolated from dogs, cats and humans; B from cats and dogs; I from cats and rabbits; O from humans and rabbits; and P from dogs and rabbits. The other genotypes were present in only one host (Table 1).

**Figure 2:**
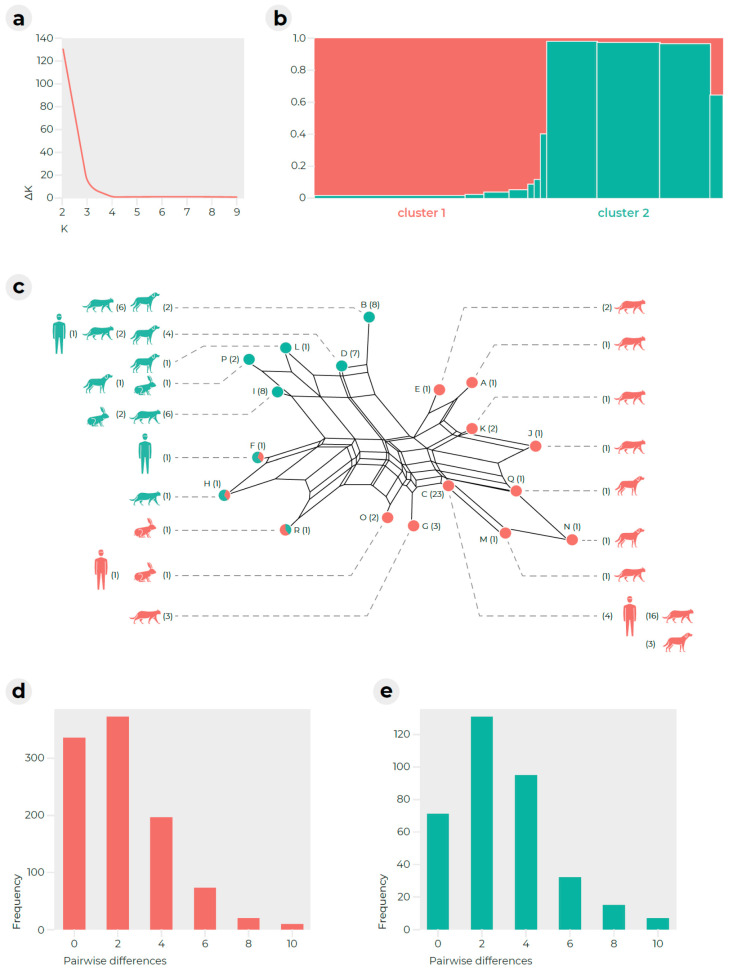
The population structure of *Microsporum canis* strains revealed by the analysis of six microsatellite loci. The population structure was examined using STRUCTURE software based on the Bayesian clustering algorithm and the peak of ΔK was observed at K = 2 (**a**). Individual strains are represented by bar plots generated in STRUCTURE that summarize Q values, i.e., the proportional membership of each individual to inferred clusters (**b**). A NeighborNet network was inferred with FAMD software and visualized in SplitsTree (**c**) using the Jaccard index-based distance matrix (Delta score = 0.08402, Q-residual score = 0.09745). The assignment of strains to clusters is indicated by red or green colour; the hosts of the individual haplotypes are indicated by icons (**c**). Histograms showing the frequency of pairwise genetic differences among individuals within populations of cluster 1 (**d**) and cluster 2 (**e**).

A Bayesian model-based clustering algorithm implemented in STRUCTURE software was used to determine how many populations were included in the dataset [15]. The highest ΔK value was observed at K = 2 (Figure 2a,b), where K represents the number of genetic groups assumed. Cluster 1 (37 strains) and cluster 2 (28 strains) contained 11 and seven genotypes, respectively. High admixture between clusters was observed in three samples corresponding to MLMT genotypes F, H and R. The ratios of strains isolated from animals with symptomatic versus asymptomatic infections were similar between clusters: 25:7 in cluster 1 and 21:5 in cluster 2 (Table S2). This distribution was not significantly different (chi-squared, *p* < 0.05). Strains from cluster 1 were mainly isolated from cats (*n* = 25), followed by dogs (*n* = 5), humans (*n* = 5) and rabbits (*n* = 2). All hosts were also included in cluster 2 but in a slightly different ratio: cats (*n* = 15), dogs (8), rabbits (3) and humans (2). There were no statistically significant differences in the distribution of hosts between the two clusters according to the chi-squared test (*p* < 0.05).

The genetic diversity indices are listed in Table S3. The low value of Nei’s gene diversity (D) (0.07 in cluster 1 and 0.12 in cluster 2) showed that the populations were genetically uniform. This low value indicated that the populations are composed of abundant clones. Random mating was rejected in clusters 1 and 2 according to the index of association I_A_, with a significance level of *p* < 0.05; I_A_ = 1.38 for cluster 1 (*p* < 0.01); I_A_ = 1.0 for cluster 2 (*p* < 0.01). Low DW index values were observed for both clusters (0.24 and 0.26, respectively), showing that these populations with their unique sets of alleles have existed over a long period of time. The frequency of pairwise differences between individuals within clusters, indicating their clonality, is shown in Figure 2d,e. Analysis of molecular variance was performed to test cluster-specific differences (Table S4) and showed that the diversity between clusters contributed a total variability of ~62%, while the diversity within clusters contributed ~38% (*p* < 0.0001). This suggested that there was a relatively high level of genetic information exchange between clusters, as also observed by the low number of fixed alleles (fixation index, F_ST_ = 0.62, *p* < 0.0001).

## 3. Discussion

The results of this study showed that *M. canis* had a low level of intraspecies variability based on the DNA markers employed for subtyping. In particular, many of the employed DNA sequence markers (i.e., *tubb*, *tef1-α*, *CaM*, *act*, *gapdh* and *mcm7*) were unable to differentiate *M. canis* strains, thus precluding these markers from being applied to track the source of infections or being used in population genetic studies in general. It was shown previously that the ITS region, *tubb* and *tef1-α* genes provided sufficient sequence variations to be useful for the differentiation of *M. ferrugineum* and *M. audouinii* from the closely related *M. canis* [16]. In this study, we confirmed these observations and broadened the spectrum of genes that are useful for differentiating among these species to also include IGS, *CaM*, *act*, *gapdh* and *mcm7* loci.

Among the 12 gene markers employed herein, only two gene markers (ITS and IGS) differentiated *M. canis* strains into five genotypes due to a single nucleotide polymorphism and indels. The discriminatory power of these loci was, however, too low. Gräser et al. [17] were the first researchers to find genetic variation between *M. canis* isolates in the ITS region and detect eight substitutions within the ITS1 and ITS2 regions. The lower level of variation reported herein probably reflected the origin of the strains from a small geographic area and a relatively short period of sampling, as previously suggested by other researchers [18,19].

The microsatellite-typing scheme that was updated in this study offers a higher discriminatory power than the MLST approaches. In this setting, *M. canis* strains were divided into 18 different genotypes with relatively high genetic diversity (Simpson’s diversity index of 0.84). This suggested that this typing scheme, which is easy and cost effective to use, may be a powerful discriminatory tool for subtyping in practice.

MLMT approaches were previously applied to 26 *M. canis* strains originating from 13 countries by Pasquetti et al. [4], who used eight markers and observed 22 genotypes. Additionally, Watanabe et al. [20] analysed 70 *M. canis* strains from Japan, 59 of which were from humans and 11 of which were from cats. The authors revealed 20 genotypes, thus confirming the high potential of microsatellite typing, as reported in our study. However, in our study, all the strains originated from one region in Italy, which probably contributed to the lower diversity detected here. In addition, we also excluded some microsatellite loci previously developed by Pasquetti et al. [4] from our typing scheme due to the presence of several motifs or interrupted repeats. Elimination of these hypervariable markers probably further reduced the observed diversity.

Using STRUCTURE software, we showed that the examined isolates belonged to two major subpopulations, i.e., cluster 1 and cluster 2. However, these populations were relatively poorly differentiated, with significant gene flow between them, as indicated by AMOVA and the relatively low number of fixed alleles in each cluster. This was also demonstrated by the presence of isolates with high admixture levels between clusters, namely, isolates CD1190, CD1192 and CD448 (MLST types F, H and R; Figure 2). There was no statistically significant difference in the distribution of hosts or symptomatic and asymptomatic animals between clusters. In conclusion, we were not able to find any link between these subpopulations and the biological characteristics of strains. This may reflect the fact that these populations were not clearly separated and were rather arbitrarily delimited. In addition, changes in the virulence level may be associated with genotypes rather than with the entire subpopulation. It may have also reflected different selection pressures that affected the studied loci and virulence factors (neutral evolution vs. positive selection).

Data from the present study showed that the MLST approach offers only very limited discriminatory power among *M. canis* strains and thus is not suitable for subtyping. Only a few markers, such as ITS and IGS regions, might be useful for the detection of limited genetic variability. In contrast, MLMT has a high discriminatory power, and the proposed typing scheme is useful for gaining insight into the dynamics of disease transmission, determining the source and routes of infections and confirming or ruling out outbreaks. In addition, MLMT might be useful for identifying virulent strains, identifying the regional and global distributions of genotype patterns and evaluating the effectiveness of control or preventive measures and interventions.

## 4. Materials and Methods

### 4.1. Source of Isolates

A total of 66 *M. canis* strains isolated from animal and human patients with dermatophytosis were employed. The strains were obtained from the Veterinary Mycology collection of the Department of Veterinary Medicine, University of Bari and all were isolated in Southern Italy.

### 4.2. Multilocus Sequence Typing (MLST)

Quick-DNA^TM^ Fungal/Bacterial Miniprep kit (Zymo research, Orange, CA, USA) was used to isolate genomic DNA from seven days old colonies grown on malt extract agar (MEA: HiMedia, Mumbai, India) as described by Hubka et al. [21]. Target loci, i.e., internal transcribed spacer region (ITS) of the rDNA, intergenic spacer region of the rDNA (IGS), the partial β-tubulin gene (*tubb*), translation elongation factor 1-α (*tef1-α*), calmodulin (*CaM*), actin (*act*), glyceraldehyde 3-phosphate dehydrogenase (*gapdh*) and minichromosome maintenance complex component 7 (*mcm7*) were amplified using primer combinations listed in Table S5. A set of eight strains from different hosts and localities was used for initial screening of intraspecies variability. *Microsporum audouinii* and *Microsporum ferrugineum* were also included to determine the applicability of these markers in distinguishing mentioned species from closely related *M. canis*.

Reaction volume (20 μL) contained 1 μL (50 ng μL^−1^) of DNA, 0.3 μL of both primers (25 pM), 0.2 μL of My Taq Polymerase and 4 μL of 5 × My Taq PCR buffer (Bioline, London, UK). PCR conditions followed previously described protocol [22]. The PCR products were visualized in an electrophoretogram (1% agarose gel with 0.5 μg mL^−1^ ethidium bromide). Automated sequencing was performed at Seqlab Sequencing Service (Charles University, Prague, Czech Republic) using both terminal primers. Obtained DNA sequences were inspected and assembled in Bioedit v. 7.0.5. The PCR reaction and DNA sequencing was repeated for samples representing rare genotypes. Alignments of genes were performed using the FFT-NS-i option implemented in MAFFT online service [23]. The unique DNA sequences were deposited into the European Nucleotide Archive (ENA) database under the numbers LR989561–LR989562, LR989270–LR989272, OU375165–OU375167, OU374853–OU374855, OU374996–OU374999, OU375053–OU375056, OU375000–OU375003, OU375004–OU375007, OU375008–OU375011, OU375012–OU375015.

### 4.3. Development of Microsatellite Markers

A BLAST (Basic Local Alignment Search Tool) search was conducted to identify microsatellite motifs using the available nucleotide sequence of *M. canis* CBS 113480 whole genome shotgun sequence (http://www.broadinstitute.org/) (accessed on 10 July 2020) using WebSat online software [24]. Thirteen loci with high number of di-, tri and tetranucleotide repeats were selected in addition to markers previously developed by Pasquetti et al. [4]. In total, 21 loci were used for further analyses. A test set of eight strains from different hosts and localities was used to ascertain presence of polymorphisms following the method of Schuelke [25]. PCR conditions were as follows: one cycle at 95 °C for 1 min; 27 cycles at 95 °C for 30 s, 55 °C for 30 s, 72 °C for 45 s, followed by eight cycles at 95 °C for 30 s, 53 °C for 30 s, 72 °C for 45 s and a final extension at 72 °C for 10 min. We checked the presence of undesirable polymorphisms in the microsatellite flanking regions and polymorphisms in the microsatellite regions by DNA sequencing using terminal primers. Interrupted repeats as well as loci containing two or more repeat motifs within the fragments were excluded. Emphasis was also placed on the selection of loci that were uniformly distributed in the available genomic sequence. Finally, six loci exhibiting some levels of polymorphism were selected for multilocus microsatellite typing (MLMT) (Table 2). Some markers (GT_13_, AC_20_–AC_14_, AT_15_, GT_15_) developed by Pasquetti et al. [4] were excluded because of the presence of interrupted repeats and some loci contained two or more repeat motifs within the fragments.

Using a Multiplex Primer Analyzer (www.thermoscientifcbio.com/webtools/multipleprimer) (accessed on 10 July 2020), primer-primer interactions were evaluated before assembling multiplexes. The forward primers of six selected loci were tagged with fluorescent dye and arranged into a single multiplex panel (Table 2). The reaction volume of 5 μL for multiplex PCR contained 1 μL DNA, 0.5 μL of water, 1 μL of the mixture of primers and 2.5 μL of Multiplex PCR Master Mix (QIAGEN, Hilden, Germany). The PCR conditions were chosen according to the manufacturer’s recommendations. The PCR products (diluted in water 1:50) were mixed with 10 μL of deionized formamide and 0.2 μL of the GeneScan™ 600 LIZ size standard (Applied Biosystems, Waltham, MA, USA) and denatured for 3 min at 95 °C, followed by analysis on an ABI 3100 Avant Genetic Analyzer in the Seqlab Sequencing Service (Charles University, Prague, Czech Republic). Peak sizes were scored with GeneMapper software and allele binning was performed with MsatAllele R package [26].

### 4.4. Statistical Analysis of Microsatellite Data

The discriminatory power of the typing scheme was calculated using Simpson’s index of diversity. A binary and allele data matrix was created using GeneMarker 3.0.1 software (SoftGenetics, LLC, State College, PA, USA) and genetic distances were calculated from the matrix and used for the construction of the NeighborNet network in the SplitsTree 4 [27]. A Bayesian model-based clustering algorithm with a clustering number K = 1–10 was applied to the allele data matrix using the software STRUCTURE [15]. Ten simulations were calculated at the www.bioportal.uio.no (accessed on 30 October 2021) server (Lifeportal, University of Oslo, Oslo, Norway) using the admixture model and 1 × 10^6^ MCMC replicates; 5 × 10^8^ replicates were discarded as burn-in. The optimal clustering number K was estimated using ΔK and similarity coefficients, [28] and both values were calculated using the script structure-sum [29] in the R version 3.3.4 [30].

The genetic variability within and between clusters was analysed via the analysis of molecular variance (AMOVA) [31] in the Arlequin [32]. The degree of gene flow among clusters was estimated using a pairwise fixation index (F_ST_) calculated in Arlequin [32]. The degree of clonality or recombination within particular clusters was estimated by calculating the index of association (I_A_) in the program MultiLocus 1.3, [33] which is used for measuring the linkage disequilibrium between alleles and is useful in inferring the occurrence of cryptic recombination in putatively asexual populations [34]. Random mating is suggested if no linkage is detected between the alleles of different loci (randomly distributed alleles); in that case I_A_, it is expected to be nearly zero or zero. We tested for significant deviation from 10,000 random multilocus permutations of genotypes under a random mating model. To measure within-population diversity, Nei’s gene diversity (D) was calculated based on the frequencies of alleles at individual loci [35,36]. The degree of genetic divergence was investigated by the rarity index (DW; frequency down-weighted marker values) [37]. All mentioned population indexes (D, DW) were calculated from binary data matrix using script AFLPdat in R 3.0.2 [30]. Frequency histograms of pairwise differences between individuals were generated using the same software.

## Figures and Tables

**Table 1:** A detailed overview of subtyping results using sequence and microsatellite markers in 66 *Microsporum canis* strains.

Sample	Source	Lesion	Typing Using ITS and IGS Loci			Multilocus Microsatellite Typing							
			ITS-GT	IGS-GT	MLST	TC_10_	GT_17_C	AG_12_	GT_17_B	CAT_8_	GT_14_	MLMT	Cluster
CD367	dog	no	G1	G1	G1	NA	NA	NA	NA	NA	NA	NA	NA
CD1131	cat	yes	G1	G1	G1	109	370	374	108	396	102	A	1
CD1133	cat	yes	NA	NA	NA	103	368	374	108	396	102	C	1
CD1134	cat	no	G1	G3	G3	103	368	374	108	396	102	C	1
CD1149	cat	yes	G1	G1	G1	103	368	374	108	396	102	C	1
CD1150	human	yes	G1	G1	G1	103	368	374	108	396	102	C	1
CD1151	cat	yes	G1	G1	G1	103	368	374	108	396	102	C	1
CD1152	human	yes	G1	G1	G1	103	368	374	108	396	102	C	1
CD1171	dog	yes	NA	NA	NA	103	368	374	108	396	102	C	1
CD1194	cat	yes	G1	G1	G1	103	368	374	108	396	102	C	1
CD1195	cat	yes	G1	G1	G1	103	368	374	108	396	102	C	1
CD1196	human	yes	G1	G1	G1	103	368	374	108	396	102	C	1
CD1211	cat	yes	G1	G1	G1	103	368	374	108	396	102	C	1
CD1233	cat	yes	G1	G1	G1	103	368	374	108	396	102	C	1
CD1595	cat	no	G1	G1	G1	103	368	374	108	396	102	C	1
CD1601	cat	yes	G1	G1	G1	103	368	374	108	396	102	C	1
CD1602	cat	yes	G1	G1	G1	103	368	374	108	396	102	C	1
CD368	dog	yes	NA	NA	NA	103	368	374	108	396	102	C	1
CD382	cat	yes	G1	G1	G1	103	368	374	108	396	102	C	1
CD396	human	yes	G1	G1	G1	103	368	374	108	396	102	C	1
CD441	dog	yes	G1	G1	G1	103	368	374	108	396	102	C	1
CD975	cat	yes	G1	G1	G1	103	368	374	108	396	102	C	1
CD976	cat	yes	G1	G1	G1	103	368	374	108	396	102	C	1
CD979	cat	yes	G1	G1	G1	103	368	374	108	396	102	C	1
CD980	cat	yes	G1	G1	G1	103	368	374	108	396	102	C	1
CD1145	cat	no	G1	G1	G1	109	368	374	108	396	102	E	1
CD1191	cat	yes	G1	G1	G1	103	368	374	108	394	102	G	1
CD383	cat	no	G1	G1	G1	103	368	374	108	394	102	G	1
CD978	cat	n0	G1	G1	G1	103	368	374	108	394	102	G	1
CD1235	cat	yes	G1	G1	G1	103	370	374	108	396	104	J	1
CD1242	cat	yes	G1	G1	G1	103	370	374	108	396	102	K	1
CD1289	cat	yes	G2	G1	G4	103	370	374	108	396	102	K	1
CD1565	cat	yes	G1	G1	G1	103	368	368	108	396	102	M	1
CD366	dog	yes	NA	NA	NA	103	368	368	108	396	104	N	1
CD384	human	yes	G1	G1	G1	103	368	374	106	396	102	O	1
CD415	rabbit	no	G1	G1	G1	103	368	374	106	396	102	O	1
CD416	dog	yes	G1	G1	G1	103	368	374	108	396	104	Q	1
CD448	rabbit	no	G1	G1	G1	107	368	374	106	394	102	R	1
CD1132	dog	yes	G1	G1	G1	105	368	374	110	396	104	B	2
CD1135	cat	yes	G1	G1	G1	105	368	374	110	396	104	B	2
CD1146	dog	yes	G1	G1	G1	105	368	374	110	396	104	B	2
CD1148	cat	yes	G1	G1	G1	105	368	374	110	396	104	B	2
CD1320	cat	yes	G1	G1	G1	105	368	374	110	396	104	B	2
CD1598	cat	yes	G1	G3	G3	105	368	374	110	396	104	B	2
CD1600	cat	yes	G1	G1	G1	105	368	374	110	396	104	B	2
CD761	cat	yes	G1	G1	G1	105	368	374	110	396	104	B	2
CD1143	human	yes	G1	G1	G1	105	368	374	110	396	102	D	2
CD1153	dog	yes	G1	G1	G1	105	368	374	110	396	102	D	2
CD1229	dog	yes	G1	G1	G1	105	368	374	110	396	102	D	2
CD1230	cat	yes	G1	G1	G1	105	368	374	110	396	102	D	2
CD1231	dog	no	G1	G1	G1	105	368	374	110	396	102	D	2
CD1232	cat	yes	G1	G1	G1	105	368	374	110	396	102	D	2
CD1567	dog	yes	G1	G1	G1	105	368	374	110	396	102	D	2
CD1190	human	yes	G1	G1	G1	107	370	374	112	396	102	F	2
CD1192	cat	no	G1	G2	G2	107	370	374	112	394	102	H	2
CD1193	cat	yes	G1	G2	G2	105	370	374	110	396	102	I	2
CD1209	cat	yes	G1	G2	G2	105	370	374	110	396	102	I	2
CD1306	cat	yes	G1	G2	G2	105	370	374	110	396	102	I	2
CD1307	cat	yes	G2	G2	G5	105	370	374	110	396	102	I	2
CD1308	cat	no	G2	G2	G5	105	370	374	110	396	102	I	2
CD409	rabbit	no	G1	G2	G2	105	370	374	110	396	102	I	2
CD412	rabbit	yes	G1	G2	G2	105	370	374	110	396	102	I	2
CD760	cat	no	G1	G2	G2	105	370	374	110	396	102	I	2
CD1279	dog	yes	G1	G1	G1	105	368	374	110	394	102	L	2
CD387	rabbit	yes	G1	G2	G2	105	370	374	110	394	102	P	2
CD430	dog	yes	G1	G2	G2	105	370	374	110	394	102	P	2

ITS-GT, ITS genotype; IGS-GT, IGS genotype; MLST, combined genotype resulting from ITS and IGS loci; MLMT, combined haplotype resulting from multilocus microsatellite typing, NA, not available (markers were were not amplified despite repeated attempts).

**Table 2:** Microsatellite markers used for multilocus microsatellite typing of *Microsporum canis* in this study.

Locus	Primer	Sequence (5′–3′)	5′-Fluorescent Dye	Product Size (bp)	Reference
AG_12_	forward	CCGAATCCCAAGAACAAGAAC	NED	368–374	this study
	reverse	CATGACCTCCAAGACCATCAC			
TC_10_	forward	TATACGATGTGTACGGCGAGAG	VIC	103–109	this study
	reverse	GTTACAGAGGAACGAACAACCC			
CAT_8_	forward	TTCAAGTCAAAGGAGAGCTGTG	PET	394–396	this study
	reverse	TGCAGTGTATTTGGGTCAAGTC			
GT_17_B	foward	GAAGGAGGTATATATGGGTGTG	NED	106–112	[4]
	reverse	GATAAGGTGTTTGGCACTGA			
GT_17_C	foward	AGGTGTTTGGCACTGAGC	VIC	368–370	[4]
	reverse	CGAAGAGAAGGAGGTATATATGG			
GT_14_	foward	GGTTTACACGCAGCATGA	PET	102–104	[4]
	reverse	CGTGGCTGAAGAAGTCTACC			

## Data Availability

The DNA sequences obtained in this study were deposited into the European Nucleotide Archive (ENA) database.

## Supplementary Materials

The following are available online at https://www.mdpi.com/article/10.3390/pathogens11010004/s1 Table S1: Accession number for various genes of *Microsporum* strains generated in this study, Table S2: Distribution of hosts and animals with and without lesion between microsatellite clusters, Table S3: Indexes of genetic diversity and cluster rarity calculated for two populations from *Microsporum* canis, Table S4: Analysis of molecular variance design and results, Table S5: List of loci screened for variability in the present study and corresponding primer pairs [38,39,40,41,42,43,44,45,46].
