# Supplementary material for: Subtyping Options for Microsporum canis Using Microsatellites and MLST: A Case Study from Southern Italy
Source: Pathogens. 2021 Dec 22;11(1):4. doi: 10.3390/pathogens11010004 (PMC8780581; doi:10.3390/pathogens11010004)
Supplement: Supplementary file 1 [file pathogens-11-00004-s001.zip › Table S1.pdf]

pathogens

MDPI

Table S1. Accession number for various genes of *Microsporum* strains generated in this study.

Species	Strain number[a]	European Nucleotide Archive (ENA) accession numbers for eight loci							
		ITS	IGS	*act*	*mcm7*	*tubb*	*CaM*	*tef1-a*	*gapdh*
M. canis	CD448	LR989561 (ITS-G1)[b]	LR989270 (IGS-G1)[b]	OU374996	OU375053	OU375000	OU375004	OU375008	OU375012
M. audouinii	CBS 404.61	OU375165	OU374853	OU374997	OU375054	OU375001	OU375005	OU375009	OU375013
M. ferrugineum	CBS 497.48	OU375166	OU374854	OU374998	OU375055	OU375002	OU375006	OU375010	OU375014
	SK 1775/19	OU375167	OU374855	OU374999	OU375056	OU375003	OU375007	OU375011	OU375015

[a] CBS, Westerdijk Fungal Biodiversity Institute (formerly Centraalbureau voor Schimmelcultures), Utrecht, Netherlands; CD, Veterinary Mycology collection, Department of Veterinary Medicine, University of Bari, Italy; SK, personal number, strain not deposited in public culture collection; [b] ITS and IGS sequences of strain CD488 were identical to CD1134 for which GenBank accession numbers were generated previously and thus not deposited again.
