# Supplementary material for: Subtyping Options for Microsporum canis Using Microsatellites and MLST: A Case Study from Southern Italy
Source: Pathogens. 2021 Dec 22;11(1):4. doi: 10.3390/pathogens11010004 (PMC8780581; doi:10.3390/pathogens11010004)
Supplement: Supplementary file 1 [file pathogens-11-00004-s001.zip › Table S2.pdf]

pathogens

MDPI

Table S2. Distribution of hosts and animals with and without lesion between microsatellite clusters.

Cluster	Total	Animals with lesion	Animals without lesion	Dog	Cat	Human	Rabbit
Cluster 1	37	25	7	5	25	5	2
Cluster 2	28	21	5	8	15	2	3
Total	65	46	12	13	40	7	5
