# Supplementary material for: Subtyping Options for Microsporum canis Using Microsatellites and MLST: A Case Study from Southern Italy
Source: Pathogens. 2021 Dec 22;11(1):4. doi: 10.3390/pathogens11010004 (PMC8780581; doi:10.3390/pathogens11010004)
Supplement: Supplementary file 1 [file pathogens-11-00004-s001.zip › Table S3.pdf]

pathogens

MDPI

Table S3. Indexes of genetic diversity and cluster rarity calculated for two populations from *Microsporum canis.*

Cluster	Number of samples	Number of genotypes	D	DW index	I_A (p value)	I_A (p value)
Cluster 1	37	11	0.071	0.24	1.38 (<0.01)	1.38 (<0.01)
Cluster 2	28	7	0.116	0.26	1 (<0.01)	1 (<0.01)

D, Nei's gene diversity; I_A, index of association
