# Supplementary material for: Subtyping Options for Microsporum canis Using Microsatellites and MLST: A Case Study from Southern Italy
Source: Pathogens. 2021 Dec 22;11(1):4. doi: 10.3390/pathogens11010004 (PMC8780581; doi:10.3390/pathogens11010004)
Supplement: Supplementary file 1 [file pathogens-11-00004-s001.zip › Table S4.pdf]

pathogens MDPI

Table S4. Analysis of molecular variance design and results.

Source of variation	Degree of freedom	Sum of squares	Variance of components	Percentage of variation
Among clusters	1	59.849	1.86380 Va	62.06
Within clusters	62	70.651	1.13953 Vb	37.94
Total	63	130.500	3.00333	
