# Supplementary material for: Subtyping Options for Microsporum canis Using Microsatellites and MLST: A Case Study from Southern Italy
Source: Pathogens. 2021 Dec 22;11(1):4. doi: 10.3390/pathogens11010004 (PMC8780581; doi:10.3390/pathogens11010004)
Supplement: Supplementary file 1 [file pathogens-11-00004-s001.zip › Table S5.pdf]

pathogens MDPI

Table S5. List of loci screened for variability in the present study and corresponding primer pairs.

Locus	Primer	Sequence (5′– 3′)	Reference
ITS	ITS1F	CTTGGTCATTTAGAGGAAGTAA	[38,39]
	NL4	GGTCCGTGTTTCAAGACGG	
IGS	LR12R	GAACGCCTCTAAGTCAGAATCC	[40]
	invSR1R	ACTGGCAGAATCAACCAGGTA	
tubb	Bt2a	GGTAACCAAATCGGTGCTGCTTTC	[41]
	Bt2b	ACCCTCAGTGTAGTGACCCTTGGC	
tef1-α	EFdermF	CACATTAACTTGGTCGTTATCG	[42]
	EFdermR	CATCCTTGGAGATACCAGC	
CaM	CF1L (forward)	GCCGACTCTTTGACYGARGAR	[43]
	CF1M (forward)	AGGCCGAYTCTYTGACYGA	
	CF4 (reverse)	TTTYTGCATCATRAGYTGGAC	
act	ACT512F	ATGTGCAAGGCCGGTTTCGC	[44]
	ACT783R	TACGAGTCCTTCTGGCCCAT	
gapdh	GPD_F	CAGGTTGTTGCTGTCAACGAC	[45]
	GPD_R	GATGTTCTGGGCAGCGGTAC	
mcm7	Mcm7-709for	ACIMGIGTITCVGAYGTHAARCC	[46]
	Mcm7-1348rev	GAYTTDGCIACICCIGGRTCWCCCAT	
